# Comparative Analysis and Development of a Flavor Fingerprint for Volatile Compounds of Vegetable Soybean Seeds Based on Headspace-Gas Chromatography-Ion Mobility Spectrometry

**DOI:** 10.3389/fpls.2021.768675

**Published:** 2021-12-14

**Authors:** Fengjie Yuan, Xujun Fu, Xiaomin Yu, Qinghua Yang, Hangxia Jin, Longming Zhu

**Affiliations:** Hangzhou Sub-Center of National Soybean Improvement, Institute of Crop and Nuclear Technology Utilization, Zhejiang Academy of Agricultural Sciences, Hangzhou, China

**Keywords:** vegetable soybean, volatile compounds, fingerprint, HS-GC-IMS, cultivals

## Abstract

Evaluating the volatile compounds and characteristic fingerprints of the core cultivars of vegetable soybean would provide useful data for improving their aroma in the breeding programs. The present study used headspace-gas chromatography-ion mobility spectrometry (HS-GC-IMS) to evaluate the volatile compounds of vegetable soybean seeds at a specific growth stage. In total, 93 signal peaks were identified, 63 compounds qualitatively, with 14 volatile flavor compounds providing multiple signals. The 63 volatile compounds consisted of 15 esters, 15 aldehydes, 13 alcohols, 15 ketones, one acid, and four other compounds. The peak intensity of most of the volatile compounds varied greatly between the core cultivars. The alcohols and aldehydes determined the basic volatile flavor of the vegetable soybean seeds. Volatile flavors were determined by their respective esters, ketones, or other components. Characteristic fingerprints were found in some core vegetable soybean cultivars. Four cultivars (Xiangdou, ZHE1754, Zhexian 65018-33, and Qvxian No. 1) had pleasant aromas, because of their higher content of 2-acetyl-1-pyrroline (2-AP). A principal component analysis (PCA) was used to distinguish the samples based on the signal intensity of their volatile components. The results showed that the composition and concentration of volatile compounds differed greatly between the core cultivars, with the volatile flavor compounds of soybeans being determined by the ecotype of the cultivar, the direction of breeding selection, and their geographical origin. Characteristic fingerprints of the cultivars were established by HS-GC-IMS, enabling them to be used to describe and distinguish cultivars and their offspring in future breeding studies.

## Introduction

Soybean (*Glycine max* L. Merr.) is the most important crop cultivated worldwide. It is a major source of protein and vegetable oil for human and animal consumption and contains several phytochemicals, such as isoflavones and phenolic compounds. Because of its high nutritional value, the soybean is processed into many different products, such as soybean flour, soybean milk, soy sauce, tofu, natto, and other snacks. The vegetable soybean (*Glycine max* L. Merr., also known as edamame) is a food-grade soybean variety that is generally harvested when the pods are fully filled and still green (Zhang and Kyei-Boahen, 2007). Vegetable soybeans are consumed widely in China, Japan, and south-east Asia as a snack food. The main edible part of the vegetable soybean is the seed, which is rich in carbohydrates, proteins, vitamins, minerals, and phytochemicals. Apart from its macronutrients and micronutrients, the dark green color of the vegetable soybean at maturity, its large seed size, soft texture, sweetness, and less beany flavor differentiate it from the regular soybean (Saldivar et al., 2011). Of these attributes, flavor often has the greatest influence on consumer acceptance and behavior. The aromatic vegetable soybean has now become more popular and gained wider acceptance in Japan, the United States, and Europe than the regular soybean (Saldivar et al., 2011). The aromatic type of vegetable soybean commands a higher price than the non-aromatic varieties in international markets, mainly because of its characteristic flavor (Wu et al., 2009). Flavor is perceived primarily by the sense of taste and olfaction (aromatics/aroma) (Glanz et al., 1998), with a unique flavor being associated with a complex mixture of compounds belonging to the different chemical classes (Bravo, 1998). Determining the diversity of these flavor compounds and their contribution to the volatile flavor of vegetable soybean seeds is invaluable for assessing the quality of the soybean at the edible stage (Castada et al., 2019).

The diversity of volatile compounds has been studied in many vegetable and fruit crops, such as rice (Monsoor and Proctor, 2004), soybean (Ramasamy et al., 2019), sorghum (Zanan et al., 2016), melon (Shi et al., 2020), pyrus (Qin et al., 2012), peppers (Ge et al., 2020), and mushroom (Li et al., 2019). The nutritional quality attributes of the vegetable soybean are mainly investigated at specific stages of seed maturity, with its aroma and overall acceptability usually evaluated by its taste (Xu et al., 2016; Jadhav et al., 2018). However, little quantitative information is available for comparing the volatile compounds of a large number of vegetable soybean cultivars at specific stages, with no information available on the composition and ratio of the volatile flavor components in vegetable soybean seeds. The present study will detect the volatile flavor compounds in boiled vegetable soybean seeds, harvested at the R6-R7 growth stage, using headspace-gas chromatography-ion mobility spectrometry (HS-GC-IMS). The effectiveness of ion mobility spectrometry (IMS) is reported as suitable for characterizing the volatile compounds because it can rapidly analyze the samples with low detection limits without pretreatment, is highly sensitive to the compounds with high electronegativity and high proton affinity, and can detect many chemically diverse compounds, such as alcohols, aldehydes, esters, and ketones (Márquezsillero et al., 2011) and has therefore become widely used in the food analysis (Arroyo-Manzanares et al., 2017).

Therefore, the present study aimed to evaluate the volatile compounds of the vegetable soybean at the core cultivar level using HS-GC-IMS. The results will provide a reference for identifying the cultivars and their offspring for improving the taste and flavor of vegetable soybeans as part of a breeding program.

## Materials and Methods

### Plant Materials

In this study, 30 vegetable soybean core cultivars from a breeding program were used. These consisted of eight spring vegetable soybean cultivars with either a high protein content or good flavor released by the Zhejiang local government; three autumn local cultivars with large-sized seeds, two of them with a green cotyledon; eight autumn vegetable soybean varieties with a different seed color and size and good flavor released in Zhejiang province; three imported vegetable soybean cultivars, representing the typical vegetable soybean flavor, to be used as core parents in the breeding program; and eight vegetable soybean breeding lines with different flavors.

All thirty cultivars (Table 1) were preserved in the Hangzhou Sub-Center of National Soybean Improvement and sown in the plots following a completely randomized block design with three replications in the experimental field of Zhejiang Academy of Agricultural Sciences, Hangzhou, Zhejiang province, China, in 2019, with each block measuring 1.5 m wide by 10 m long. The orchard management procedures, such as fertilization and irrigation, were same for all the cultivars. The fresh soybean pods were harvested at the R6-R7 stage, with 50 pods collected from each vegetable cultivar block and combined for each of the three replicates. The fresh soybean pod samples were wrapped in aluminum foil then stored at −80°C until the subsequent analyses.

**TABLE 1:** The ecotype and origin of the 30 vegetable soybean cultivars used in the present study.

No	Cultivars	Ecotype/origin	No	Cultivars	Ecotype/origin
1	Zheqiudou No. 5	Autumn/Zhejiang	16	ZH716	Spring/Zhejiang
2	Zheqiudou No. 2	Autumn/Zhejiang	17	ZK1754	Spring/Zhejiang
3	Zhechun No. 3	Spring/Zhejiang	18	Taiwan 75	Spring/Taiwan
4	Zhechun No. 4	Spring/Zhejiang	19	TMD	Spring/Zhejiang
5	Zhechun No. 8	Spring/Zhejiang	20	Zhexian No. 12	Spring/Zhejiang
6	Xiangdou	Autumn/Shanghai	21	Zhexian No. 21	Spring/Zhejiang
7	Danbo black	Autumn/Japan	22	Zhexian No. 9	Spring/Zhejiang
8	Qingpiqingren	Autumn/Zhejiang	23	Zhexian 19	Spring/Zhejiang
9	Lvpiqingren	Autumn/Zhejiang	24	Zhexian 2013	Spring/Zhejiang
10	Kaixinlv	Summer/Liaoning	25	Zhexian 77	Spring/Zhejiang
11	Xiaonongqiuyan	Autumn/Zhejiang	26	Zhexian 76004	Spring/Zhejiang
12	Qvxian No. 1	Autumn/Zhejiang	27	Zhexian 6-12	Spring/Zhejiang
13	Zhexian No. 84	Autumn/Zhejiang	28	Zhexian 65018-18	Spring/Zhejiang
14	Zhexian No. 85	Autumn/Zhejiang	29	Zhexian 65018-32	Spring/Zhejiang
15	Huning95-1	Spring/Shanghai	30	Zhexian 65018-33	Spring/Zhejiang

### HS-GC-IMS Data Acquisition

The soybean pod samples were first boiled in water at 100°C for 5–8 min, then saved for subsequent analyses using a FlavourSpec gas chromatograph (G.A.S. Gesellschaft für Analytische Sensorsysteme mbH, Dortmund, Germany), equipped with a CombiPal GC autosampler (CTC Analytics AG, Zwingen, Switzerland).

The soybean seed samples (3 g) were placed in 20-ml headspace vials, incubated at 60°C for 15 min spinning at 500 rpm and then, a headspace volume of 400 μl was automatically injected by a syringe at 65°C into an MXT-5 capillary column (15 m, i.d. 0.53). Nitrogen (99.99% purity) was used as the carrier gas programmed as follows: initial flow rate of 2 ml/min, maintained for 2 min, increased to 100 ml/min over 18 min, then maintained at 100 ml/min for 2 min before stopping. The analytes were separated in the column at 60°C then ionized in the IMS ionization chamber at 45°C, with a constant gas flow of 150 ml/min. Furthermore, 2-ketones were used to standardize the instrument as the IMS was not responsive to the alkanes. The 2-ketones (C4-C9) were used to calculate the retention index (RI) of the volatile compounds as an external reference. The volatile compounds were identified by comparing their RI values using the Gas Chromatographic Retention Database in NIST and the Drift Time Database (self-established). The total analysis time was about 20 min.

### Data Analysis

All the experiments were performed three times. All data were acquired in the positive ion mode, with each spectrum formed from an average of 12 scans. Three software programs developed by G.A.S were used to view the analytical spectrum and for quantitative analysis. During the first step, the Laboratory Analytical Viewer (v.2.2.1, G.A.S.) and Reporter analysis (v.1.2.12, G.A.S.) were used to compare the 2D top view, 3D spectrogram, and the spectral differences among the samples. In addition, a GC × IMS Library Search (v.1.0.3, G.A.S.) was used for the qualitative analysis of the volatile of compounds based on their retention time in the GC column and drift time (time of flight in the drift tube). The reference RI data were supplied by NIST 2014, and the drift time data by G.A.S. The quantitative analysis was based on the peak height of the selected signal peak using the gallery plot analysis (v.1.0.7, G.A.S.). A principal component analysis (PCA) was used to visualize the differences between the soybean cultivars with Dynamic PCA software (G.A.S.).

A standard curve was established between the peak height and a 2-acetyl-1-pyrroline (2-AP) standard sample (99%). The content of 2-AP in the vegetable soybean cultivars was calculated by using the external standard method.

## Results

### Analysis of HS-GC-IMS Spectra in Vegetable Soybean Seeds

**FIGURE 1:**
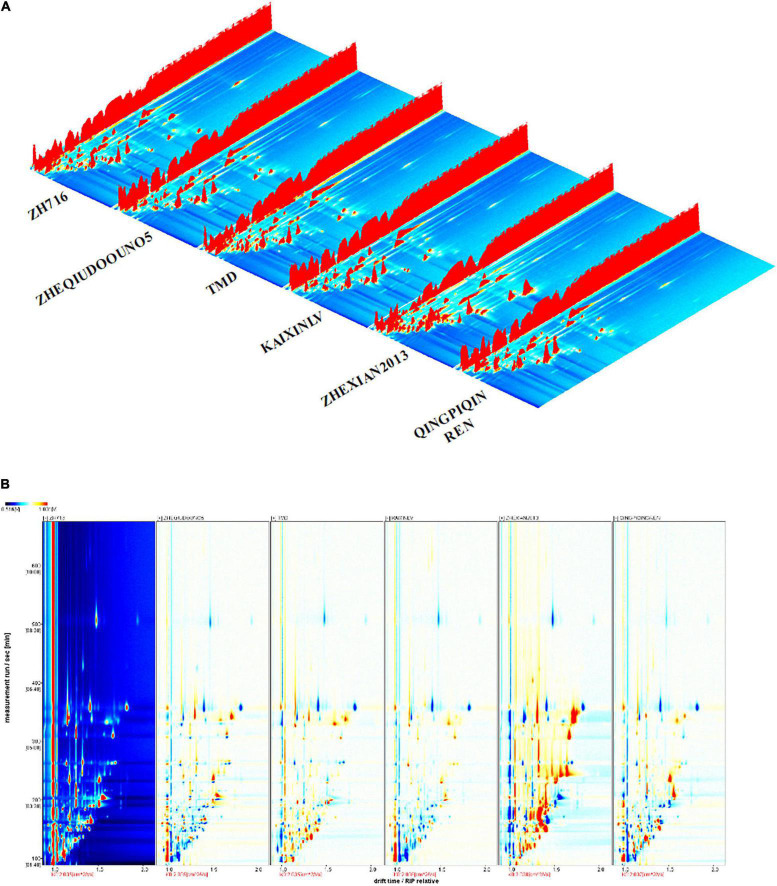
Changes in the volatile compounds from the seeds of six representative vegetable soybean cultivars shown in three-dimensional (3D) **(A)** and two-dimensional (2D) **(B)** topographic forms.

Headspace-gas chromatography-ion mobility spectrometry three-dimensional (3D) and two-dimensional (2D) spectra were used to analyze the changes and diversity of the volatile flavor compounds of the vegetable soybean samples. The data are represented using a 3D topographical visualization and 2D topographic plot. The differences between the different cultivars were obvious (Figures 1A,B). In Figure 1A, the *X*-axis represents the ion drift time of the volatile flavor compounds, the *Y*-axis represents the gas phase retention time, and the *Z*-axis represents the peak intensity. The peak signal distributions of the different samples were very similar, but the signal intensity varied, indicating that the content of volatile flavor compounds differed among the samples.

In a further comparison using 2D spectra (Figure 1B), the reactive ion peak (RIP) is represented by the red vertical line at the horizontal coordinate of 1.0, with each point on the right side of RIP representing the type of volatile compound, with the retention time of most signals appearing between 100 and 600 s. To compare the differences among the samples, sample ZH716 was used as a reference, with the spectral background of the other samples being subtracted from this reference. After subtraction, the background was white, with the red area indicating that the content of the compound was higher than that of the reference sample, and the blue area indicating that it was lower. Figure 1B shows the diversity of volatile flavor compounds among the different samples directly.

### Qualitative Analysis of Volatile Flavor Compounds in Vegetable Soybean Seeds by HS-GC-IMS

**FIGURE 2:**
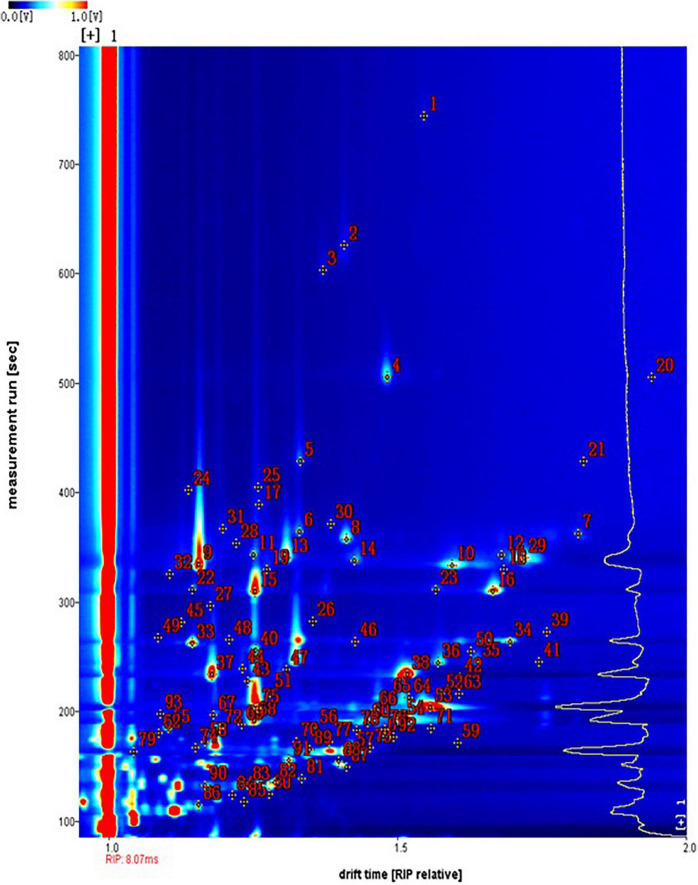
The topographic plots of gas chromatography-ion mobility spectrometry (GC-IMS) spectra obtained from 30 vegetable soybean cultivars. The numbers 1–93 correspond to the detected signals, with those identified listed in Table 2 and the other numbers representing unidentified signals.

The spectral topographic plots of the Huning 95-1 cultivar were selected for the qualitative analysis, because all the samples had similar volatile flavor compounds. In Figure 2, each dot represents the type of volatile flavor compound. Most of the dots were concentrated in the range of retention times between 100 and 400 s and abscissae between 1 and 2. The compounds identified were numbered, with unmarked dots denoting unidentified compounds. From all the samples, 93 signal peaks were identified and 63 compounds were qualitatively identified using the built-in NIST database and IMS database in the GC-IMS library search (Table 2). Fourteen volatile flavor compounds provided multiple signals, such as monomers and dimers. These included methyl octanoate, ethyl hexanoate, ethyl 2-methylpropanoate, ethyl propanoate, (E)-2-octenal, (E)-hept-2-enal, (E)-2-hexen-1-ol, oct-1-en-3-ol, pentan-1-ol, *trans-*2-pentenal, 6-methyl-hept-5-en-2-ol, 3-octanone, 1-octen-3-one, and 2-heptanone. These compounds exhibited similar retention times, but different migration times, related to their content. In the ionization region, the protonated molecules and neutral molecules combined to form dimers, whose quantity could be enhanced by a high content of the compounds (Ewing et al., 1999; Arroyo-Manzanares et al., 2017; Rodríguez-Maecker et al., 2017).

**TABLE 2:** Gas chromatography-ion mobility spectrometry (GC-IMS) integration parameters of the volatile compounds detected in the 30 vegetable soybean cultivars used in the present study.

No.	Compounds	CAS N0	Molecule formula	RT	RI	DT
1	Ethyl nonanoate	C123295	C11H22O2	744.318	1,279.2	1.54734
2	2-Heptylfuran	C3777717	C11H18O	625.734	1,195.8	1.40878
3	(E,Z)-2,6-Non-adienal	C557482	C9H14O	603.054	1,179.9	1.373
4	Methyl octanoate-M	C111115	C9H18O2	505.221	1,111.0	1.48412
5	(E)-2-Octenal-M	C2548870	C8H14O	428.289	1,057	1.33344
6	Ethyl hexanoate-M	C123660	C8H16O2	364.378	1,012.1	1.33189
7	Ethyl hexanoate-D	C123660	C8H16O2	362.127	1,010.5	1.8143
9	Oct-1-en-3-ol-M	C3391864	C8H16O	334.082	986.8	1.15717
10	Oct-1-en-3-ol-D	C3391864	C8H16O	333.043	985.5	1.59629
11	6-Methyl-hept-5-en-2-ol-M	C1569604	C8H16O	343.538	997.4	1.25248
12	6-Methyl-hept-5-en-2-ol-D	C1569604	C8H16O	342.717	996.9	1.6822
13	3-Octanone-M	C106683	C8H16O	340.256	994.2	1.30858
15	(E)-Hept-2-enal-M	C18829555	C7H12O	309.572	957.3	1.25503
16	(E)-Hept-2-enal-D	C18829555	C7H12O	309.244	956.9	1.66689
18	1-Octen-3-one-D	C4312996	C8H14O	330.305	982.2	1.68612
19	1-Octen-3-one-M	C4312996	C8H14O	330.125	982	1.27522
20	Methyl octanoate-D	C111115	C9H18O2	505.549	1,111.3	1.94189
21	(E)-2-Octenal-D	C2548870	C8H14O	428.453	1,057.1	1.82428
25	Benzene acetaldehyde	C122781	C8H8O	404.794	1,040.5	1.26018
29	3-Octanone-D	C106683	C8H16O	340.656	994.7	1.72139
30	Ethyl hex-3-enoate-M	C2396830	C8H14O2	371.285	1,017	1.38579
31	(E, E)-2,4-Hheptadienal	C4313035	C7H10O	367.182	1,014.1	1.19904
32	3-Furanmethanol	C4412913	C5H6O2	325.076	957.9	1.10743
33	3,4-Dimethylthiophene	C632155	C6H8S	262.175	900.3	1.14686
34	Heptanal	C111717	C7H14O	263.459	901.8	1.69677
35	n-Hexanol	C111273	C7H14O	245.481	873.7	1.64108
37	(E)-2-Hexen-1-ol-M	C928950	C6H12O	233.41	851.4	1.18027
38	(E)-2-Hexen-1-ol-D	C928950	C6H12O	233.41	851.4	1.51857
39	Amyl acetate	C628637	C7H14O2	272.705	913	1.76081
40	2-Hheptanone-M	C110430	C7H14O	255.754	892.6	1.25684
41	Isoamyl acetate	C123922	C7H14O2	245.224	873.3	1.74689
43	Ethyl 2-methylbutanoate	C7452791	C7H14O2	227.76	840.9	1.24292
44	(Z)-3-hexen-1-ol	C928961	C6H12O	239.528	862.7	1.23289
45	2-Acetyl-1-pyrroline	C85213225	C6H9NO	281.577	923.6	1.12706
46	Allylacetic acid	C591800	C5H8O2	263.539	901.9	1.42771
49	3-Methylthiopropanal	C3268493	C4H8OS	267.253	906.4	1.08724
50	2-Heptanone-D	C110430	C7H14O	255.376	892.1	1.62874
53	Hexanal-D	C66251	C6H12O	203.58	796.1	1.558
54	Pentan-1-ol-D	C71410	C5H12O	192.428	772.3	1.51051
55	*Trans-*2-pentenal-M	C1576870	C5H8O	184.531	752.4	1.10452
56	*Trans-*2-pentenal-D	C1576870	C5H8O	184.472	752.3	1.3591
57	Pentanal	C110623	C5H10O	163.531	699.5	1.42268
61	2-Methylbutan-1-ol	C137356	C5H12O	177.233	734	1.47685
67	2-Hexanone	C591786	C6H12O	196.843	783.4	1.18353
68	Pentan-1-ol-M	C71410	C5H12O	191.273	769.4	1.25306
69	Isobutyl acetate	C110190	C6H12O2	188.204	761.7	1.23193
70	3-Hydroxybutan-2-one	C513860	C4H8O2	171.939	720.7	1.32652
71	Ethyl 2-methylpropanoate-D	C97621	C6H12O2	184.199	751.6	1.56015
72	Ethyl 2-methylpropanoate-M	C97621	C6H12O2	184.812	753.1	1.19454
73	Ethyl propanoate-D	C105373	C5H10O2	166.611	707.3	1.45352
74	Ethyl propanoate-M	C105373	C5H10O2	167.122	708.6	1.15153
75	Hexanal-M	C66251	C6H12O	203.055	795.1	1.25879
79	1-Propene-3-methylthio	C10152768	C4H8S	163.218	698.7	1.04449
80	Butanal	C123728	C4H8O	124.157	566.5	1.27936
81	Ethyl Acetate	C141786	C4H8O2	139.053	618.4	1.33626
83	2-Butanone	C78933	C4H8O	132.102	594.2	1.24322
87	3-Methylbutanal	C590863	C5H10O	149.341	654.2	1.41376
88	2-Methylbutanal	C96173	C5H10O	154.609	672.6	1.40052
89	3-Pentanone	C96220	C5H10O	165.145	703.6	1.35003
90	2,3-Butanedione	C431038	C4H6O2	132.369	595.1	1.16847
91	1-Penten-3-one	C1629589	C5H8O	154.995	674	1.31331
92	3-Methylbutan-1-ol	C123513	C5H12O	176.127	731.2	1.49496
93	2,3-Hexanedione	C3848246	C6H10O2	197.37	784.6	1.09121

*-D, dimer; -M, monomer.*

### Relative Abundance of Major Volatile Flavor Compounds in the Core Vegetable Soybean Cultivars

A total of 63 diverse volatile compounds were identified by using the GC-IMS, consisting of esters (15), aldehydes (15), alcohols (13), ketones (15), acid (1), and other volatiles (4). The relative peak signal volumes of the volatile compounds from the different cultivars are given in a Supplementary Table. The results showed that the peak volumes of most of the volatile compounds varied greatly between the different cultivars.

Of the 15 ester compounds, acetyl esters (9/15), such as ethyl acetate, ethyl hexanoate, ethyl hex-3-enoate, ethyl 2-methylpropanoate, ethyl 2-methylbutanoate, and ethyl nonanoate were predominant. Among the ester compounds, the peak signal of ethyl acetate with its rich content ranged most widely, from 121.98 to 8,003.02 a.u., followed by amyl acetate, methyl octanoate-M and ethyl hexanoate-D, with peak volumes ranging from 13.59 to 1,268.59 a.u., 176.96 to 1,946.78 a.u., and 88.03 to 1,056.93 a.u., respectively. These four ester compounds might contribute to the diversity of the ester notes in the aromas of the 30 vegetable cultivars. Ethyl nonanoate exhibited the narrowest range of peak volumes of all the ester compounds from 52.93 to 136.94 a.u.

Hexanal-D and oct-1-en-3-ol-M had the richest content, with a range of peak volumes of 1,344.21–8,611.99 a.u. and 4,223.95–8,436.49 a.u., respectively.

Ketones, which contribute to the flavor of food, were detected in the vegetable soybean seeds. Compared with the alcohols and aldehydes, the peak volumes of most ketone compounds (10/15) were less than 1,000 a.u. The highest peak volumes of the ketone compounds were for 3-octanone-D and 3-octanone-M, followed by 3-pentanone and 2-butanone. As ester compounds, the ketones in the vegetable soybean seeds provided the characteristic volatile flavor of the different vegetable soybean cultivars.

Five other compounds were evaluated in the present study: allylacetic acid, 1-propene-3-methylthio, 3,4-dimethylthiophene, 2-heptylfuran, and 2-acetyl-1-pyrroline. Then, 2-acetyl-1-pyrroline was detected and quantified by the external reference method. This showed that four cultivars had a higher content of 2-AP: Xiangdou, ZHE1754, Zhexian 65018-33, and Qvxian No. 1 (Table 3).

**TABLE 3:** 2-acetyl-1-pyrroline (2-AP) content in 30 different vegetable soybean cultivars.

No.	Cultivals	Content of 2-AP (μg/g)	No.	Cultivals	Content of 2-AP (μg/g)
1	Zheqiudou No. 5	–	16	ZH716	–
2	Zheqiudou No. 2	–	17	ZK1754	7.21 ± 0.62
3	Zhechun No. 3	–	18	Taiwan 75	–
4	Zhechun No. 4	–	19	TMD	–
5	Zhechun No. 8	–	20	Zhexian No. 12	–
6	Xiangdou	6.04 ± 0.08	21	Zhexian No. 21	–
7	Danbo black	–	22	Zhexian No. 9	–
8	Qingpiqingren	–	23	Zhexian 19	–
9	Lvpiqingren	–	24	Zhexian 2013	–
10	Kaixinlv	–	25	Zhexian 77	–
11	Xiaonongqiuyan	–	26	Zhexian 76004	–
12	Qvxian No. 1	6.33 ± 1.97	27	Zhexian 6-12	2.02 ± 0.62
13	Zhexian No. 84	–	28	Zhexian 65018-18	–
14	Zhexian No. 85	–	29	Zhexian 65018-32	2.07 ± 0.14
15	Huning95-1	–	30	Zhexian 65018-33	10.76 ± 1.85

### Fingerprints of Cultivars Based on Volatile Substances Using HS-GC-IMS

**FIGURE 3:**
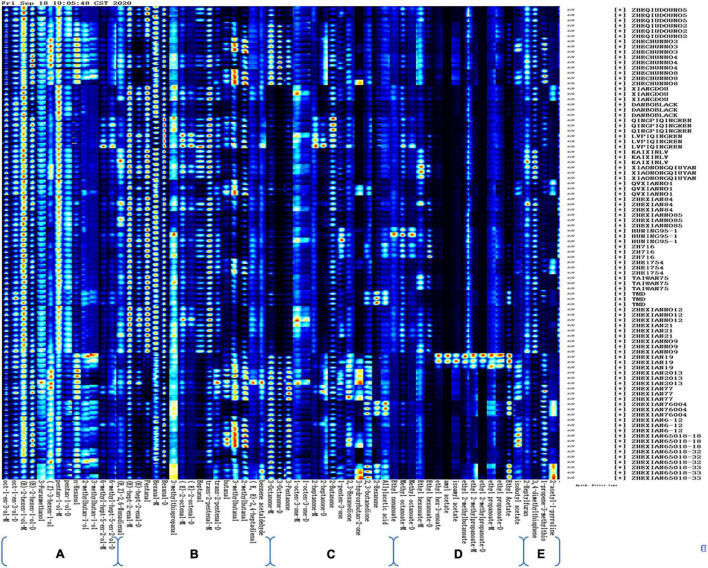
Gallery plot of the signal peak areas obtained from 30 vegetable soybean cultivars. **(A)** Alcohol compounds; **(B)** aldehyde compounds; **(C)** ketone compounds; **(D)** ester compounds; and **(E)** other compounds.

The volatile compounds in the different vegetable soybean cultivars were analyzed by using the HS-GC-IMS. Fingerprint imaging (Figure 3) shows the gallery plot of each sample and their color differences so that the content of volatile compounds can be approximated by the color of each square, with a brighter color representing a higher content of the compound. Each column indicates a signal peak, and each row represents a sample, with three sample repeats. Ninety-three compounds were detected in the 30 samples, with 63 compounds being analyzed qualitatively and quantitatively. Of the 63 compounds, pentan-1-ol, (E)-2-hexen-1-ol, (E)-hept-2-enal, oct-1-en-3-ol, and hexanal were detected and had a comparatively high content in all 30 samples. In contrast, ethyl hex-3-enoate, amyl acetate, isoamyl acetate, and ethyl 2-methylpropanoate-D were not detected in the 30 samples except for Zhexian No. 19. The seeds of this cultivar exhibited the nine strongest signal peaks for ester compounds: ethyl acetate, amyl acetate, ethyl hex-3-enoate-M, ethyl 2-methypropanoate-D, ethyl 2-methypropanoate-M, ethyl 2-methylbutanoate, ethyl propanoate-D, isoamyl acetate, and ethyl propanoate-M. Therefore, these peak signals constituted a very particular fingerprint feature for the Zhexian No. 19 cultivar (Figure 3).

The strongest peak signals for ethyl nonanoate, methyl octanoate-D, and methyl octanoate-D were provided by the Huning 95-1 cultivar, therefore, these three volatile flavor compounds could be taken as marker signals for the Huning 95-1 cultivar. The characteristic fingerprint of the Lvpiqingren cultivar consisted of strong peak signals for (E)-2-octenal-D, (E)-2-octenal-M, heptanal, and *trans-*2-pentenal-M. Of the 13 alcohol compounds, the peak signals of (Z)-3-hexen-1-ol, pentan-1-ol-D, and n-hexanol provided a special fingerprint for the Zhechun No. 3, Zhechun No. 4, and Zhechun No. 8 cultivars, which all have a high protein content. The Lvpiqingren and Qingpiqingren cultivars exhibited stronger peak signals for 6-methyl-hept-5-en-2-ol-D and 6-methyl-hept-5-en-2-ol-M than the other cultivars. These two cultivars were summer ecotypes and local germplasms with green cotyledons, an exceptional phenotype compared with the other cultivars.

Ten ketones and one acid were detected in the present study, with the peak signals of 2,3-butanedione, 2-hexanone, and allylacetic acid endowing the characteristic fingerprints for the Zhexian76004 and TMD cultivars. These three compounds exhibited strong peak signals in the Zhexian 65018-33 cultivar, as well as the strongest peak signal for 3-hydroxybutan-2-one. The Lvpiqingren cultivar exhibited a special peak signal for 2-heptanone-D, 2-heptanone-M, and 2-butanone. Of the five other volatile compounds, the peak signal for 2-acetyl-1-pyrroline showed the brightest color from the Zhexian 65018-33 cultivar.

### PCA Analysis Based on Volatile Substances Detected by HS-GC-IMS

**FIGURE 4:**
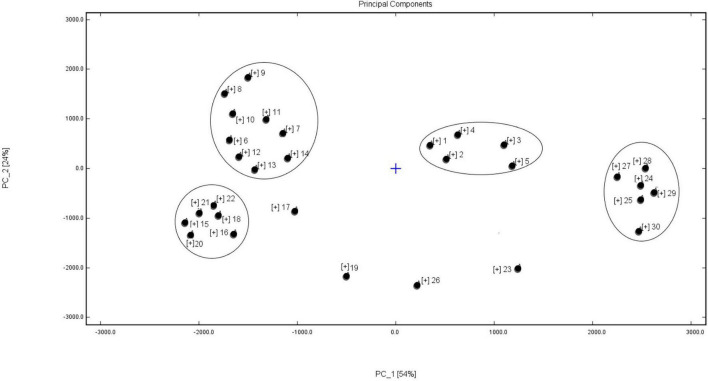
Principal component analysis (PCA) results for volatile flavor compounds from vegetable soybean seeds from 30 different cultivars. The numbers, 1–30, indicate the 30 soybean cultivars shown in Table 1.

Principal component analysis can reduce the number of dimensions and classify the original data. Figure 4 shows that the two principal components explained 78% of the total variance: PC1 54% and PC2 24%. The data from the present study were separated into four groups. Group I, consisting of five high-protein soybean cultivars, were poorly correlated with the other vegetable soybean cultivars. The autumn and summer ecotype vegetable soybean cultivars were gathered in the same region to form group II. The spring ecotype vegetable soybean cultivars divided into the groups III and IV, with the flavors between these two groups varying greatly. The six cultivars in group III included Taiwan 75 and Huning 95-1, which were introduced from the Shanghai City and Taiwan regions and were one of the hybrid parents of the other four cultivars. In group IV, six cultivars were bred by crossing local soybean cultivars with cultivars from Japan. Four cultivars, ZK1754, TMD, Zhexian No. 19, and Zhexian 76004, belonged to a single category, because of their special volatile flavor components, which was consistent with the results on the special fingerprints of volatile flavor.

## Discussion

### HS-GC-IMS Analytical Approach for the Measurement of Volatile Compounds in Plant-Based Products

Several analytical approaches are developed for measuring the volatile compounds in plant-based products. GC-MS has been considered a powerful analytical instrument to identify the chemical. However, time-consuming, complex sample pretreatment, and a significant constraint in the distinguishing of isomeric molecules, particularly ring-isomeric compounds (Kranenburg et al., 2020) limit its application for the plant-based products screening. GC-O-MS is a powerful tool for extracting aroma-active compounds from the complex mixtures, because of repetitive time-consuming labor, this method is not ideal for the rapid detection of volatile organic compounds (VOCs) in the plant-based products (Wang et al., 2020). Despite the fact that gas sensors are sensitive at room temperature and have strong selectivity and low detection limits, their detection performances are greatly influenced by moisture and air fluctuation in the environment, resulting in unstable results and poor instrument repeatability (Chen et al., 2018, 2020).

Ion mobility spectrometry is an emerging technique for detecting the trace gases and for characterizing chemical ionic substances based on the differences in the rate of migration of gas phase ions in an electric field (Li et al., 2019). This technique has many advantages over other methods, such as a rapid speed of detection, high sensitivity, easy preparation, and simple sample preparation steps. However, there are also some limitations for IMS, especially for the complex samples (Arce et al., 2014). Combining IMS with GC column could provide a better method for detecting the volatile compounds and has already proved to be useful for analyzing and characterizing the volatile compounds. HS-GC-IMS has been widely and usefully applied for analyzing wine, eggs, jujube fruits, and honey (Garrido-Delgado et al., 2011; Cavanna et al., 2019; Sun et al., 2019; Wang et al., 2019). More importantly, HS-GC-IMS can also be used to establish the visual fingerprints of volatile compounds which show changes in their variety and progress during a food process (Ge et al., 2020). This could be the most powerful function of GC-IMS compared with other analysis methods.

### Volatile Compounds in Vegetable Soybean

Various volatile compounds may serve as indicators of the maturity of soybeans and biochemical markers to evaluate seed quality. Several classes of compounds, such as alcohols, aldehydes, esters, and ketones, were the main volatile compounds identified in the present study. Of these compounds, acetyl esters were the main characteristic components, agreeing with the previous studies reporting that esters with fruity notes were the major aromatic components found in the ripe fruit (Lara et al., 2003; Moyaleon et al., 2006; Qin et al., 2012). Aldehydes and alcohols are C6 volatile compounds, often referred to as “green leaf” volatiles because of their green grass note (Yang et al., 2009), and were found to be important background volatiles of vegetable soybeans in the present study. Some previous studies have reported that the light and water characteristics and growth temperatures can significantly affect the formation of volatile metabolites, particularly alcohols and aldehydes in the plants (Bertrand et al., 2012; Benelli et al., 2015). In addition, the present study has found that some alcohols and aldehydes in vegetable soybean seeds exhibited significantly different peak volumes, even though these cultivars were produced in the same environment. These results indicated that the genetic background of the cultivar was another major factor determining the volatile flavor metabolites.

The previous studies have reported that the major volatiles of grain soybeans were ethanol, 1-octen-3-ol, phenylethyl alcohol, hexanal, octanal, 2-propanone, and r-butyrolactone (Lee and Shibamoto, 2000; Boué et al., 2003; Dings et al., 2005; Kim et al., 2020). However, most of these compounds were not detected in the present study except for oct-1-en-3-ol and hexanal, possibly because of the different sampling stages and different soybean processing methods. In the present research, the vegetable soybean seed samples were harvested at the R6 growth stage then the fresh pods were boiled in water. The specific harvesting stage and treatment method also contributed to the presence of some particular volatile compounds. In contrast, the different extraction methods used by HS-GC-IMS could be another factor affecting the composition of volatile flavor compounds. Two other important compounds were found in the present study, furans and 2-acetyl-1-1-pyrroline (2-AP). Furans usually exhibit sweet, burned, and baking odors formed through Maillard reactions (Fischer et al., 2017), and showed a stable peak volume among the cultivars so could have contributed to the basic flavor of the vegetable soybean. 2-acetyl-1-1-pyrroline (2-AP), a 5-membered *N*-heterocyclic ring compound, is identified as the most important compound contributing to the aroma of soybean (Wu et al., 2009; Arikit et al., 2011a,b). It was also detected in the six cultivars where its content was significantly higher than that in the other cultivars, a result consistent with their taste and aroma.

Overall, the soybean cultivars had stronger alcohol and aldehyde component peak signals, but also possessed special ester and ketone signals. In other words, the alcohol and aldehyde compounds determined the basic volatile flavor of the vegetable soybean seeds, with particular volatile flavors being determined by their respective compositions of esters, ketones, or other compounds.

The most abundant volatile flavor compound signals were detected in the spring vegetable soybean cultivars, the Zhexian serial cultivars from Zhexian 12 to Zhexian 65018-33 (Figure 4). The autumn soybean cultivars, Zheqiudou No. 2 and Zheqiudou No. 5, exhibited the most similar volatile flavor compound signals. A summer vegetable soybean cultivar, such as Kaixinlv, and some imported vegetable soybean cultivars, such as Taiwan 75, exhibited fairly abundant flavor compound signals. These results could contribute in forming the breeding objectives, and select the direction of breeding and growing environment. The quality objectives for breeding spring vegetable soybean varieties were good taste, flavor, and shape, with the better aromas provided by individual or lines of cultivars being selected by breeders during the selection procedure. In contrast, Zheqiudou No. 2 and Zheqiudou No. 5 were bred for producing the soy food products, such as tofu, soy sauce, and soybean milk, with little attention being paid to the flavor of the fresh seed. Most summer and autumn cultivars were intended for use as vegetables and for producing soy foods, so the flavor of the fresh soybean seeds was given only moderate consideration. However, the growing environment for all the cultivars would have some effect on their volatile flavor compounds.

The use of volatile-compound imaging and determining the markers based on HS-GC-IMS for discriminating among the vegetable soybean cultivars was a non-targeted approach to analysis. This could play an important role in screening for the specific markers, and for extracting reliable, unbiased, and visual information from a large amount of data (Wang et al., 2019). The present study found that some specific fingerprints belonged to different cultivars, that the content of volatile flavor compounds in the different soybean cultivars could be affected by their cultivation method or growing environment, but that some particular volatile flavors (specific fingerprints) could be determined by special genes that could be passed on to their offspring. Therefore, these types of data visualization combined with the results of sensory evaluation could be applied for selecting the volatile flavors and breeding new vegetable varieties with pleasant and acceptable flavors.

### PCA Analysis of Volatile Compounds in Vegetable Soybean

A PCA is a multivariate statistical analysis method that linearly transforms several variables to select a smaller number of significant variables (Li et al., 2019). The PCA results on volatile compounds from the 30 vegetable soybean cultivars tended to show a clear separation. The soybean cultivars with a high seed protein content were less correlated with the other vegetable soybean cultivars. The volatile flavor compounds of these high-protein soybean cultivars were different to the middle or lower protein content soybean cultivars. The autumn and summer ecotype vegetable soybean cultivars were located in the same PCA region, thus reflecting the higher correlation of volatile flavor components of these samples. The flavors of the spring ecotype vegetable soybean cultivars varied greatly.

The particular volatile flavor compounds were determined by the ecotype of the cultivars, the direction of breeding selection, and the original cultivation area. The data from HS-GC-IMS contained useful information and could be a reasonably useful tool for distinguishing among the vegetable soybean cultivars. These non-targeted characteristic markers offer the potential for selecting the new vegetable soybean lines with good volatile flavors in the future breeding programs.

In conclusion, 93 volatile compounds were detected in the seeds from 30 vegetable soybean cultivars, of which 63 compounds were detected qualitatively. Alcohol and aldehydes were the predominant volatile compounds followed by esters and ketones. The composition and concentration of volatile compounds differed greatly between the cultivars.

Some characteristic fingerprints of vegetable cultivars, established using HS-GC-IMS, could be used to describe and distinguish cultivars and their offspring in the future studies. Four vegetable cultivars that exhibited an aromatic flavor because of their high 2-AP content would be valuable in the vegetable soybean breeding programs. Based on the PCA analysis, the volatile flavor compounds of the soybean seeds were determined by using the ecotype of the cultivars, the direction of breeding selection, and the geographical origin.

## Data Availability Statement

The original contributions presented in the study are included in the article/Supplementary Material, further inquiries can be directed to the corresponding author/s.

## Author Contributions

XF participated in planting vegetable soybean cultivars. XY performed the statistical analysis and helped to draft the manuscript. HJ collected the materials. QY helped for the analysis of the data. LZ helped to perform the HS-GC-IMS experiment. FY designed the study, carried out the HS-GC-IMS experiment and helped in drafting the manuscript. All authors have read and approved this manuscript.

## Conflict of Interest

The authors declare that the research was conducted in the absence of any commercial or financial relationships that could be construed as a potential conflict of interest.

## Publisher’s Note

All claims expressed in this article are solely those of the authors and do not necessarily represent those of their affiliated organizations, or those of the publisher, the editors and the reviewers. Any product that may be evaluated in this article, or claim that may be made by its manufacturer, is not guaranteed or endorsed by the publisher.
